# Identification of the essential protein domains for Mib2 function during the development of the *Drosophila* larval musculature and adult flight muscles

**DOI:** 10.1371/journal.pone.0173733

**Published:** 2017-03-10

**Authors:** Katrin Domsch, Andreas Acs, Claudia Obermeier, Hanh T. Nguyen, Ingolf Reim

**Affiliations:** Department of Biology, Division of Developmental Biology, Friedrich-Alexander University of Erlangen-Nürnberg, Erlangen, Germany; University of Valencia, SPAIN

## Abstract

The proper differentiation and maintenance of myofibers is fundamental to a functional musculature. Disruption of numerous mostly structural factors leads to perturbations of these processes. Among the limited number of known regulatory factors for these processes is Mind bomb2 (Mib2), a muscle-associated E3 ubiquitin ligase, which was previously established to be required for maintaining the integrity of larval muscles. In this study, we have examined the mechanistic aspects of Mib2 function by performing a detailed functional dissection of the Mib2 protein. We show that the ankyrin repeats, in its entirety, and the hitherto uncharacterized Mib-specific domains (MIB), are important for the major function of Mib2 in skeletal and visceral muscles in the *Drosophila* embryo. Furthermore, we characterize novel *mib2* alleles that have arisen from a forward genetic screen aimed at identifying regulators of myogenesis. Two of these alleles are viable, but flightless hypomorphic *mib2* mutants, and harbor missense mutations in the MIB domain and RING finger, respectively. Functional analysis of these new alleles, including *in vivo* imaging, demonstrates that Mib2 plays an additional important role in the development of adult thorax muscles, particularly in maintaining the larval templates for the dorsal longitudinal indirect flight muscles during metamorphosis.

## Introduction

Building and maintaining muscles is a crucial aspect for the fitness of most metazoan organisms. Mutations that lead to improper muscle development or function often affect genes that encode proteins with a complex domain structure and multiple functions; hence they are difficult to dissect in complex organisms with long generation times. *Drosophila melanogaster* is a valuable model organism for uncovering such genes and investigating particular domain-specific functions. Indeed, mutation- or RNAi-induced muscle defects can be detected efficiently in genetic screens, and transgenes of various forms of interest can be introduced into different genetic backgrounds with relatively little effort for subsequent analysis (for examples see [1–5]). Although far from complete, we currently have a good understanding of how larval and adult skeletal (somatic) muscles are built in the *Drosophila* embryo and during metamorphosis, respectively (for recent reviews see [6, 7]). Each fiber of the larval body wall musculature is generated by fusion of a particular founder cell, which imparts muscle-specific identity, with several fusion-competent myoblasts [8–10]. As the nascent fibers grow, they target their attachment sites provided by epidermal tendon cells, and eventually differentiate into mature muscle fibers with a regular array of myofibrils [7, 11]. During metamorphosis, most larval muscles are histolyzed and replaced by new adult muscles, thereby allowing the organism to shift from larval crawling locomotion to the more complex movements of adult flies. The adult flight musculature encompasses a set of relatively small direct flight muscles (steering muscles) and several large indirect flight muscles (IFMs), which are attached to the thoracic cuticle and are responsible for generating the major contracting force during wing movement. The IFMs are subdivided into two antagonistic stretch-activated muscle sets: one set consisting of three bilateral bundles of dorso-ventral muscles (DVMs), whose contraction results in upward movement of the wing, and a second set of six pairs of dorsal longitudinal muscles (DLMs), which are required for downward wing movements [12–14]. DVMs, like adult abdominal muscles and leg muscles, are generated *de novo* from founder cells and fusing myoblasts, both of which are generated from a population of undifferentiated cells known as adult muscle precursors (or AMPs) that are set aside during embryogenesis. By contrast, DLMs are generated via fusion of the AMPs to larval muscle templates, three bilateral pairs of thoracic oblique muscles that are protected from metamorphosis-induced histolysis during pupal stages [15, 16].

While much emphasis has been put on elucidating the early embryonic events of myogenesis, less is known about the regulatory aspects that enable maturation and stabilization of muscles after initial myotubes have been formed. The gene *mind bomb2* (*mib2*) was previously identified as a muscle founder cell-specific gene that is essential for maintaining muscle integrity [17, 18]. In *mib2* null mutants, well-formed muscles undergo apoptotic cell death and progressively detach from myotendinous junctions during the final stages of embryonic development. The Mib2 protein possesses a complex domain organization that is shared with Mib1, including a RING finger with presumed E3 ubiquitin ligase activity [19–22]. In addition to two C-terminal RING fingers, Mib2 contains two Mib-HERC2 (or HERC2) domains that flank a ZZ-type zinc finger domain, a tandem repeat of Mind bomb-specific sequences (hereafter referred to as MIB domains) and a stretch of ankyrin repeats. This domain structure is generally conserved in vertebrate Mind bomb proteins, Mib1 and Mib2 (a.k.a. Skeletrophin), which like *Drosophila* Mib1 have been implicated in the processing of Notch ligands [23–25], although their exact role *in vivo* is not fully understood. Notably, published data indicate that the muscle maintenance function of *Drosophila* Mib2 is unrelated to Notch signaling processes because Mib1 and another Notch ligand-ubiquitinating ligase, Neuralized, are both unable to substitute for Mib2; Notch-related processes such as specification of fusion-competent myoblasts are also not affected in *mib2* mutants [17, 18]. Furthermore, the two RING finger domains, which are presumed to be associated with E3 ubiquitin ligase activity, are dispensable for muscle maintenance, although they contribute to the robustness of muscle fusion events [17, 18].

To date, the role of the other Mib2 protein domains in muscle formation has not been evaluated. Here we present our analysis of specific Mib2 protein deletions and show the individual contributions of each of these domains to the development of somatic and gut muscles in the embryo. We also describe four novel EMS-induced *mib2* alleles that cause either strong null-like muscle defects or lead to specific hypomorphic effects. Of note, the hypomorphic alleles have allowed us to characterize the additional specific requirement of *mib2* for the formation of adult flight muscles.

## Materials and methods

### *Drosophila* stocks

The following stocks were used: *mib2*^*1*^ [17]; *mib2*^*S2616*^, *mib2*^*S0768*^, *mib2*^*S1259*^, and *mib2*^*S1456*^ (this study); *rP298-Gal4* (provided by S.D. Menon, Institute of Molecular and Cell Biology, Singapore; [26]); *Df(2L)Exel8039*, *Df(2L)ED1202* and several additional deficiencies spanning Chromosome 2 (all available from the Bloomington Stock Center at Indiana University, USA). The *mib2* mutant stocks were maintained over the *SM6b*, *eve-LacZ*, *CyO*, *twi>eGFP* or *CyO*, *Kr>GFP*^*S65T*^ balancer.

### Generation of *mib2* deletion constructs and rescue experiments

*mib2* deletion constructs were generated by amplifying the region of interest from the EST LP14687 (obtained from the Berkeley *Drosophila* Genome Project), and the resulting PCR-generated fragments were cloned into the pUAST vector with Eco RI and Kpn I restriction sites and confirmed by sequencing. Details about the primers will be provided upon request. Embryo injections were subsequently performed by the BestGene company (Chino Hills, CA, USA).

The established transgenic lines are designated as: *UAS-mib2*^*FL*^ (Full length: spanning aa 1–1049, nt 1–3150), *UAS-mib2*^Δ*HERC2A+ZZ*^ (deleting 109 aa HERC2A and ZZ domains: nt 22–345), *UAS-mib2*^Δ*ZZ*^ (deleting 36 aa ZZ domain: nt 238–345), *UAS-mib2*^Δ*HERC2B*^ (deleting 74aa HERC2B domain: nt 466–651), *UAS-mib2*^Δ*MIB*^ (deleting 144aa MIB domain: nt 757–1188), *UAS-mib2*^Δ*ANK*^ (deleting 314 aa ANK domain: nt 1387–2301), *UAS-mib2*^Δ*N-termANK*^ (deleting 168 aa N-terminal portion of ANK domain: nt 1387–1746), and *UAS-mib2*^Δ*C-term*ANK^ (deleting 146 aa C-terminal portion of ANK domain: nt 1759–2301) constructs.

For analysis, *mib2*^*1*^*/SM6b*, *eve-LacZ; rP298-Gal4* virgin females were crossed with males from each of the “testing” transgenic stocks (*mib2*^*1*^*/SM6b*, *eve-LacZ* with the above listed full-length or modified *UAS-mib2* construct). The progeny were then assessed for the degree of rescue that was conferred by each type of “testing” construct.

### Identification of new *mib2* mutant alleles

Novel *mib2* alleles were isolated from an EMS mutagenesis screen for genes affecting myogenesis using tissue-specific RFP and GFP reporters, as described in Hollfelder et al. (2014) [4]. Presumptive strong and weak *mib2* alleles were categorized, primarily based on the presence of late embryonic somatic muscle defects that were either severe (rounding or disappearance of many muscle fibers, including the *org-1*-RFP-labeled abdominal muscle #5/LO1) or sporadic and subtle (rounding of a subset of LO1 fibers). The strong alleles *mib2*^*S0768*^ and *mib2*^*S2616*^ were found to be lethal *in trans* with each other and *mib2*-deleting deficiencies *Df(2L)ED1202* and *Df(2L)Exel8039* as well as with the documented null allele *mib2*^*1*^ [17]. Homozygous escapers of the sub-viable alleles *mib2*^*S1259*^ could be recovered and maintained as a homozygous stock. For the *mib2*^*S1456*^ allele, viability- and fertility-reducing portions of the mutant *S1456* chromosome, including the *w*^*+*^ or *y*^*+*^-labeled RFP/GFP screening transgenes that were initially present, needed to be removed by recombination before it could be maintained as a homozygous stock.

The presence of *mib2* mutations was established by sequencing *mib2* exon-spanning DNA fragments, which were PCR-amplified using DNA from manually sorted *twi*>eGFP-negative homozygous embryos or adults (if viable). All mutations were verified by sequencing two independent DNA preparations. DNA from the non-mutagenized RFP/GFP reporter strains *S-18a-13b-16c*.*1* and *S-18a-13b-16b*.*1*, as described in Hollfelder et al. (2014), served as “EMS control” reference [4].

### Immunocytochemistry of whole mount embryos

Collections enriched for late embryonic stages were used. The embryos were de-chorionated, fixed, and stained, as described in Nguyen and Xu (1998) [27]. The following specific antibodies were used: anti-Tropomyosin 1 (MAC 141; 1:400; Babraham Institute, UK), rabbit anti-β3 Tubulin (1:3000; provided by R. Renkawitz-Pohl; U. of Marburg, Germany), mouse anti-βPS Integrin (1:10; Developmental Studies Hybridoma Bank, USA), guinea pig anti-Mib2 (1:200; provided by Marta Carrasco-Rando) [18]. Fluorescent secondary antibodies were used at 1:200 (Dianova, Germany).

The stained embryos were analyzed on a Zeiss ApoTome microscope with 20x/0.8 Plan-Apochromat and 40x/1.3 Plan-Apochromat Oil objectives. Images were captured and analyzed with the Axiovision 4.8 program, followed by further processing with Adobe Photoshop.

### Analysis of adult thoracic muscles

Hemi-thoraces were prepared and fixed in 3.7% formaldehyde/ PBS, as described in Domsch et al. (2013), prior to being stained with Phalloidin-Atto 550/ PBS/ 0.1%Tween-20 (1:3000; Sigma-Aldrich, Germany) [28]. After extensive washes in PBT (PBS/ 0.1% Tween-20), the stained hemi-thoraces were mounted in Vectashield (Linaris, Germany) for subsequent analysis on a Zeiss ApoTome microscope.

For a more detailed analysis, sectioning of the thoraces was performed. In this case, entire thoraces were first prepared and fixed overnight at 4°C in 2.5% formaldehyde/ 5% glutaraldehyde/ PBS. Following several washes in 1x PBT (PBS/ 0.1% Tween-20), the fixed thoraces were stained overnight at 4°C with Phalloidin-Atto 550/ PBS (1:3000; Sigma-Aldrich). Afterwards, the stained thoraces were again washed in 1x PBT and then placed in a 2% agarose solution. After polymerization, 80μm sections were obtained with a vibratome (Leica VT 1000s). The sections were mounted in Vectashield (Linaris, Germany) and analyzed on a Zeiss ApoTome microscope with a 20x/0.8 Plan-Apochromat objective.

### Live imaging of adult myogenesis in pupae

White pre-pupae carrying an X-chromosomal *Mhc-tau*::*GFP* insertion (Chen et al., 2003; obtained from F. Schnorrer, Max-Planck-Institute of Biochemistry) were collected with a brush and briefly rinsed with tap water. Then, they were mounted in two rows near the edges of a piece of 12 mm-wide double-stick tape with the anterior spiracles still exposed to air after being covered with a small amount of halocarbon oil and a 12x12 mm cover slip. Time-lapse image series were acquired in parallel from mutants and the non-mutagenized EMS control. Approximately four pupae were monitored per session, using the "mark and find" mode of a Leica SP5 II confocal system equipped with a HC PL APO 10x/0.40 CS dry objective and an argon laser (excitation at 488 nm, detection at 500–550nm). Acquisition was done over a time course of about ~60–70 hours at 22°C, corresponding to pupal stages P3 to P8 (according to [29]). The following settings were used: scan speed of 200 Hz; no averaging; resolution of 1024 x 520 pixel; pinhole of 1.2–1.4; Z-stack of 25–32 sections with a step size of 7–8μm; and time intervals of 5 minutes per stack. Imaging did not have any visible effects on development as all imaged pupae reached late pharate stages. Movies were generated using the Leica Application Suite Advanced Fluorescence (LAS-AF) 2.4.1 and ImageJ 1.4 programs.

### Flight tests

Adult progeny from each EMS control and mutant genotypes were tested for their flying capability. Multiple groups of utmost 10 flies each were gently pushed into a two-liter graduated cylinder and the number of fliers (hovering at the top) versus non-fliers (remaining at the bottom) was recorded.

## Results

### Expression of domain-specific deletion variants of Mib2 uncovers critical functions of the MIB domains and ankyrin repeats in maintaining muscle fibers and supporting midgut morphogenesis in the embryo

**Fig 1:**
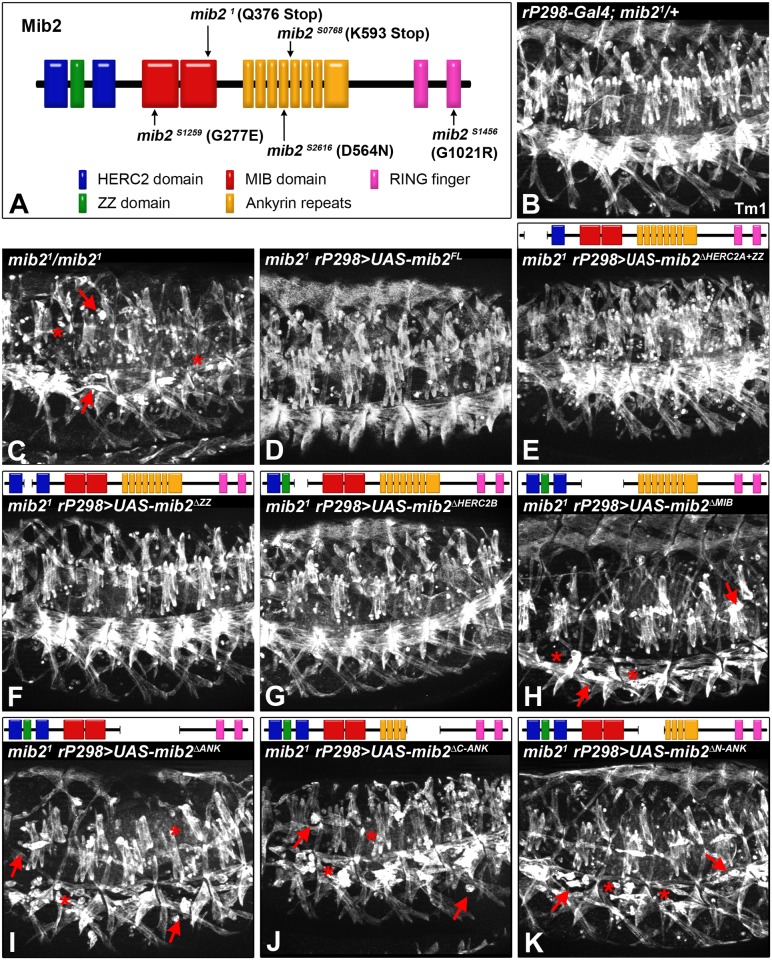
The MIB domains and all ankyrin repeats, but not HERC2 and ZZ domains, are critical for Mib2 function during somatic muscle maturation. (A) Schematic diagram of the Mib2 protein structure is shown with the different domains: HERC2 domains (blue); ZZ domain (green); MIB domains (red); ankyrin repeats (orange); RING fingers (pink). The location and type of mutation that is associated with *mib2* alleles described in subsequent sections is indicated. Muscle-specific expression of *UAS-mib2* constructs, each harboring a particular deletion, was achieved with the *rP298-Gal4* driver. Late stage 16 embryos were stained with an antibody against Tropomyosin 1 (Tm1) and analyzed by fluorescent microscopy. (B) Heterozygous *mib2* embryo with one copy of the driver *rP298-Gal4* shows a stereotypic muscle pattern. (C) Homozygous *mib2* mutant embryo shows extensive muscle loss (asterisks) and detachment (arrows). Homozygous *mib2* mutant embryos are rescued with *UAS-mib2*^*FL*^ (D; encoding full-length Mib2), *UAS-mib2*^Δ*HERC2A+ZZ*^ (E; Mib2 without the HERC2A and ZZ domains), *UAS-mib2*^Δ*ZZ*^ (F; Mib2 without the ZZ domain) or *UAS-mib2*^Δ*HERC2B*^ (G; Mib2 without the HERC2B domain). By contrast, a significant number of missing and detaching muscles is observed in homozygous *mib2* mutant embryos with *UAS-mib2*^Δ*MIB*^ (H; Mib2 without the MIB domain), *UAS-mib2*^Δ*ANK*^ (I; Mib2 without all ankyrin repeats), *UAS-mib2*^Δ*C-ANK*^ (J; Mib2 without the C-terminal ankyrin repeats) *or UAS-mib2*^Δ*N-ANK*^ (K; Mib2 without the N-terminal ankyrin repeats).

The Mib2 protein contains multiple recognizable domains, some of which have been found in proteins that are involved in ubiquitination and protein degradation pathways (i.e. HERC2 domain, RING fingers; see Fig 1A), while others are presumed to function in protein-protein interactions (i.e. ZZ domain, ankyrin repeats) [30–32]. Published studies have only demonstrated that the terminal RING fingers are not essential for mediating the main function of *mib2* in maintaining the integrity of the mature musculature, although they play a role in fusion-related events at an earlier stage [17, 18]. To assess the contribution of the other domains to Mib2 function, we generated *UAS* constructs, which encode Mib2 proteins with a deletion of a specific domain, and used the *rP298-Gal4* driver to express the altered Mib2 proteins in *mib2* mutant embryos. For analysis, we stained the embryos with an antibody against Tropomyosin 1 (Tm1) to mark the musculature and to determine the potential of each construct to rescue the *mib2* mutant phenotype. When compared to wild-type controls, the full-length *mib2* construct (*UAS-mib2*^*FL*^) is able to restore a normal somatic muscle pattern in *mib2* mutants harboring this construct (compare Fig 1D to 1B and 1C). Interestingly, the construct *UAS-mib2*^Δ*HERC2A+ZZ*^ or *UAS-mib2*^Δ*ZZ*^, encoding a Mib2 protein that is missing the first HERC2 plus ZZ domain or only the ZZ domain, respectively, is also able to restore a relatively normal somatic musculature (Fig 1E and 1F). Only a limited number of unfused myoblasts is apparent in mutant embryos with the *UAS-mib2*^Δ*HERC2A+ZZ*^ construct, as compared to the extensive muscle loss and detachment in *mib2* mutant embryos (Fig 1C). The construct *UAS-mib2*^Δ*HERC2B*^ coding for a Mib2 protein with a deletion of only the second HERC2 domain is capable of rescuing *mib2* mutants to a similar degree as the aforementioned constructs (Fig 1G). Counting the number of intact somatic muscle fibers at the end of embryogenesis (n = 25 hemi-segments for each genotype) resulted in equal numbers of fibers in heterozygous control embryos and homozygous *mib2* mutant embryos that express either the full-length *UAS-mib2* construct or *UAS-mib2*^Δ*HERC2A+ZZ*^, *UAS-mib2*^Δ*ZZ*^ or *UAS-mib2*^Δ*HERC2B*^ (S1 Fig).

The HERC2/ZZ domains are followed by two “MIB domains”, which correspond to two repeated sequence blocks that are found in all mouse, zebrafish and *Drosophila* Mib proteins [17, 24, 33]. Although highly conserved, no specific role has yet been ascribed to these sequences. We show herein that the MIB domains are indeed important for Mib2 function. The *mib2* mutant phenotype is poorly rescued with the *mib2*^Δ*MIB*^ construct, as a phenotype characterized by missing or detaching muscles was observed in mutant embryos that carry this construct (compare Fig 1H to 1B and 1C). The average number of maintained muscles is significantly reduced when compared to heterozygous *mib2*^*1*^ controls; however, comparison with homozygous null mutants suggests that this construct can provide some residual functionality (see S1 Fig). We also analyzed the importance of the ankyrin repeats that are located in the distal portion of the protein. The *mib2*^Δ*ANK*^ construct with a deletion of the entire ankyrin repeats does not provide effective rescue as mutant embryos harboring this construct exhibit severe muscle defects that are comparable to those in *mib2* mutants (compare Fig 1I to 1C, see also S1 Fig). To determine whether some ankyrin repeats could be superfluous, we also tested constructs in which either the carboxy-terminal (*mib2*^Δ*C-ANK*^) or amino-terminal (*mib2*^Δ*N-ANK*^) portion of the ankyrin repeats was deleted. Of note, the degree of ineffective rescue by either construct is similar to the ineffective rescue by the construct lacking all ankyrin repeats (compare Fig 1J and 1K to 1I, see also S1 Fig).

**Fig 2:**
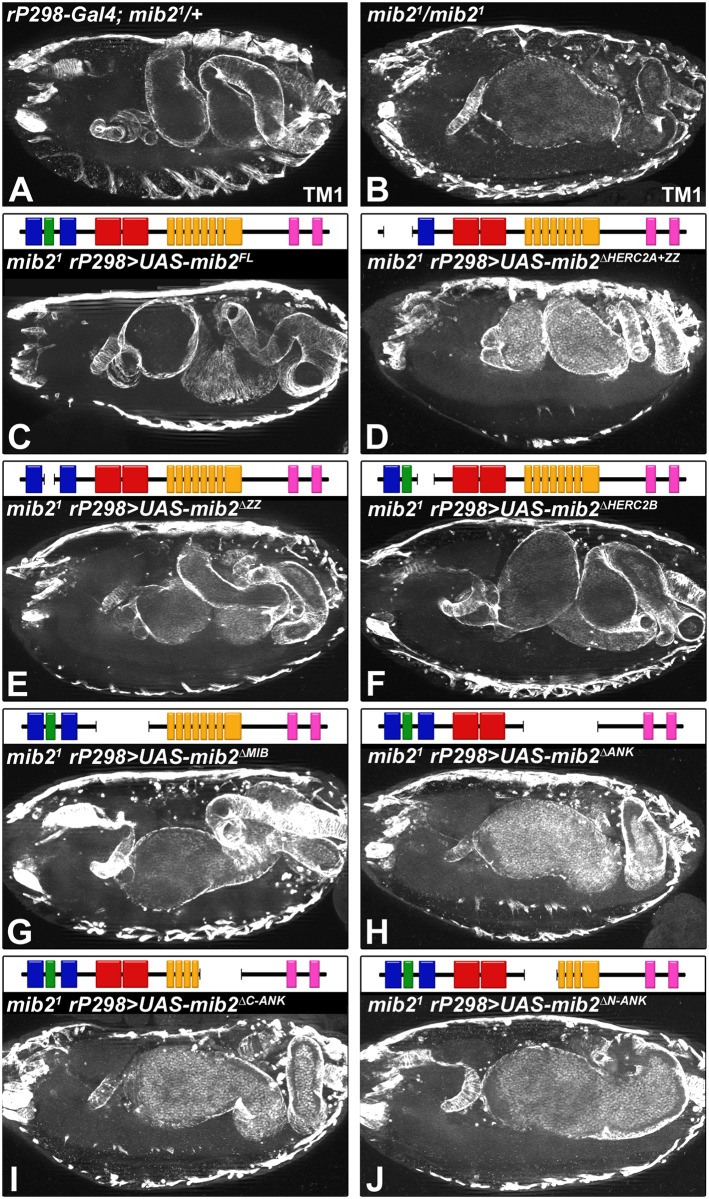
Normal midgut development depends on the MIB domains and all ankyrin repeats. Muscle-specific expression of *UAS-mib2* constructs, each with a particular deletion, was achieved with the *rP298-Gal4* driver. Late stage 16 embryos were stained with an antibody against Tropomyosin 1 (Tm1) and analyzed by fluorescent microscopy. For each indicated genotype, the examples shown herein are representative for all embryos (n≧5). (A) Heterozygous *mib2* embryo with one copy of the driver *rP298-Gal4* shows a well-formed midgut with proper constrictions. (B) Homozygous *mib2* mutant embryo exhibits a characteristic “bloated” midgut without constrictions. Normal midgut formation and morphogenesis is restored in homozygous *mib2* mutant embryos with *UAS-mib2*^*FL*^ (C; encoding full-length Mib2), *UAS-mib2*^Δ*HERC2A+ZZ*^ (D; Mib2 without the HERC2A and ZZ domains), *UAS-mib2*^Δ*ZZ*^ (E; Mib2 without the ZZ domain) or *UAS-mib2*^Δ*HERC2B*^ (F; Mib2 without the HERC2B domain). A”bloated”, unconstricted midgut is observed in homozygous *mib2* mutant embryos lacking the MIB-specific domain (*UAS-mib2*^Δ*MIB*^ in G) or portions of the ankyrin repeat (*UAS-mib2*^Δ*ANK*^ in H, *UAS-mib2*^Δ*C-ANK*^ in I and *UAS-mib2*^Δ*N-ANK*^ in J).

We had previously documented that Mib2 function is also required for proper development of the midgut musculature ([17]; see also Fig 2B). To gain insights into the mechanism, we used the same rescue constructs as above to test for the relative importance of the various domains in mediating Mib2 function during midgut development. For this analysis, we also used the anti-Tropomyosin (Tm1) antibody to mark the visceral musculature. As seen in Fig 2, mutant embryos carrying the full-length *mib2* construct (*UAS-mib2*^*FL*^) show normal midgut formation and morphogenesis when contrasted with control embryos (compare Fig 2C to 2A). Mutant embryos with the *UAS-mib2*^Δ*HERC2A+ZZ*^, *UAS-mib2*^Δ*ZZ*^ or *UAS-mib2*^Δ*HERC2B*^ construct also exhibit a well-formed midgut with proper constrictions (Fig 2D–2F). By contrast, a “bloated” midgut lacking any visible constrictions is observed in mutant embryos that harbor the *UAS-mib2*^Δ*MIB*^ construct (Fig 2G). Moreover, a similar “bloated midgut” phenotype is also associated with mutant embryos that carry the *mib2*^Δ*ANK*^ construct, which is missing all ankyrin repeats (Fig 2H), or constructs lacking a specific portion of the ankyrin repeats, such as *mib2*^Δ*C-ANK*^ and *mib2*^Δ*N-ANK*^ (Fig 2I and 2J). In the aggregate, the data show that the ZZ and HERC domains in Mib2 are not critical for its function during embryonic midgut development. As observed with the somatic musculature, the MIB domains and the region spanning the ankyrin repeats are also needed for proper midgut development.

Collectively, the data from the rescue experiments show that the ZZ domain is dispensable for the major muscle maintenance functions of Mib2 in the embryo. The HERC2 domain also appears to be non-essential for this aspect, although the consequences of deleting both HERC2 domains need to be examined to rule out functional redundancy of these domains. For the MIB domains, we showed that the deletion of a fragment containing both MIB domains abolishes most of the embryonic Mib2 function. In this assay, we did not test the possibility that either one of the two domains on its own might be dispensable in the embryo. Our analysis also suggests that the ankyrin repeats in their entirety are requisite for Mib2 function in maintaining a stable somatic musculature and supporting morphogenesis of the midgut musculature during embryogenesis.

### Characterization of four novel *mib2* alleles

**Fig 3:**
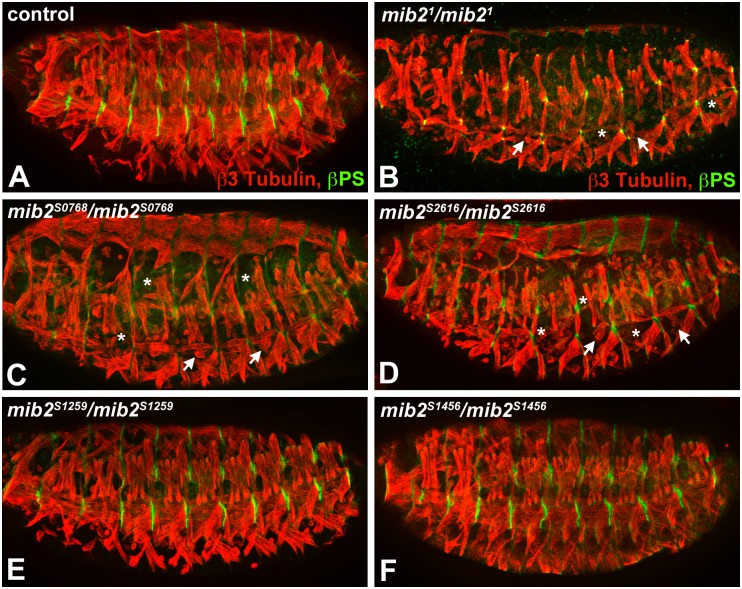
*mib2*^*S0768*^ and *mib2*^*S2616*^ mutant embryos have severe somatic muscle defects. (A-F) Late stage embryos were double-labeled with antibodies against β3-Tubulin (red) and βPS integrin (green) to visualize the muscles and attachment sites, respectively. As compared to the control embryo (A), missing (asterisks) or detaching (arrows) muscles are readily detectable in homozygous *mib2*^*1*^ (B), *mib2*^*S0768*^ (C), and *mib2*^*S2616*^ (D) mutant embryos. Homozygous *mib2*^*S1259*^ (E) and *mib2*^*S1456*^ (F) mutant embryos exhibit a normal somatic musculature.

Fortuitously, four new distinct *mib2* alleles were obtained from a recent genetic screen for mutations that affect muscle development (for details see Materials and methods). Based upon complementation data and initial reporter gene-based phenotypic analysis, *mib2*^*S0768*^ and *mib2*^*S2616*^ were classified as stronger alleles, while *mib2*^*S1456*^ and *mib2*^*S1259*^ were considered as weaker alleles (data not shown). To analyze the embryonic phenotype in greater detail, control and mutant embryos were double-stained with antibodies against β3-Tubulin and βPS integrin to visualize the muscles and attachment sites, respectively. As documented previously, homozygous *mib2*^*1*^ mutant embryos exhibit a defective somatic musculature that is characterized by a striking loss of muscles and detaching muscles (compare Fig 3B to 3A; [17]). Analogously, homozygous *mib2*^*S0768*^ and *mib2*^*S2616*^ mutants also show a severe embryonic mutant phenotype, including a significant loss of muscles and a large number of detaching muscles (Fig 3C and 3D). The staining pattern of βPS integrin is similarly disturbed in *mib2*^*1*^, *mib2*^*S0768*^, and *mib2*^*S2616*^ mutant embryos. In contrast to these severe muscle defects, we observe a normal pattern of β3-Tubulin stained muscles and well-defined βPS integrin-stained attachment sites in *mib2*^*S1259*^ and *mib2*^*S1456*^ mutant embryos, although a very limited number of detaching muscles are detectable in these mutants (Fig 3E and 3F and data not shown).

**Fig 4:**
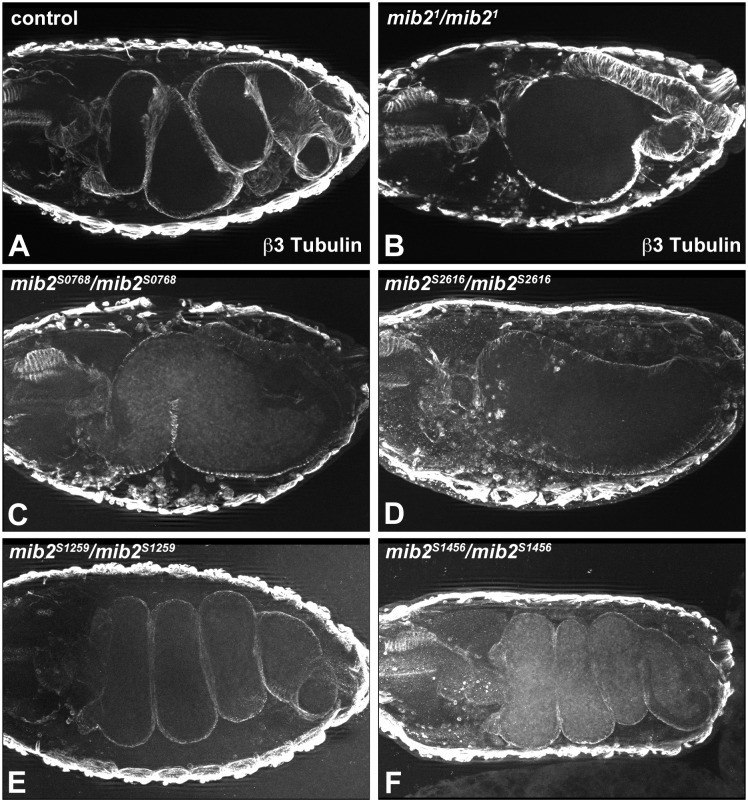
*mib2*^*S0768*^ and *mib2*^*S2616*^ mutant embryos exhibit abnormal midgut development. Late stage 16 embryos were stained with an antibody against β3-Tubulin. In contrast to the control embryo (A), homozygous *mib2*^*1*^ (B), *mib2*^*S0768*^ (C), and *mib2*^*S2616*^ (D) mutant embryos show a “bloated” midgut with abnormal constrictions. Normal constriction and morphogenesis are however observed in homozygous *mib2*^*S1259*^ (F) and *mib2*^*S1456*^ (G) mutant embryos.

For further characterization, we compared visceral muscle development in the published *mib2*^*1*^ versus the new *mib2* alleles. As reported earlier, *mib2*^*1*^ mutant embryos show a “bloated” midgut that lacks the characteristic constrictions (compare Fig 4B to 4A; [17]). Notably, homozygous *mib2*^*S0768*^ and *mib2*^*S2616*^ mutant embryos also exhibit aberrant midgut development, including an absence of proper constrictions (Fig 4C and 4D). An irregular arrangement of the longitudinal visceral muscle fibers was observed with *HLH54F-RFP* in the initial screen procedure (data not shown). These mutant phenotypes were also confirmed in trans-heterozygous *mib2*^*0768*^*/mib2*^*1*^ and *mib2*^*2616*^*/mib2*^*1*^ embryos (data not shown). In contrast to these severe defects, the midgut in *mib2*^*S1259*^ and *mib2*^*S1456*^ mutant embryos is well formed, showing normal midgut morphogenesis (Fig 4E and 4F).

The severe somatic muscle and midgut defects that are associated with the *mib2*^*S0768*^ and *mib2*^*S2616*^ alleles are consistent with the complementation data, which show that they behave as stronger alleles than the *mib2*^*S1259*^ and *mib2*^*S1456*^ alleles. To attempt to correlate the type of molecular lesion and the mutant phenotype, sequencing of DNA from the non-mutagenized parental strains (EMS controls) and the new mutant alleles was undertaken. Previously, it was established that a nonsense mutation in *mib2*^*1*^ yields a mutant protein devoid of all ankyrin repeats and RING fingers ([17]; see also Fig 1A). The present sequencing analysis revealed that *mib2*^*S0768*^ also contains a nonsense mutation, although the truncated mutant protein only lacks the carboxyl-terminal portion of the ankyrin repeats and the RING fingers (Fig 1A). This mutation resembles the *mib2*^*4*^ allele, which was previously noted as also being a functional null allele [17]. Interestingly, missense mutations were identified in *mib2*^*S2616*^, *mib2*^*S1456*^, and *mib2*^*S1259*^. In *mib2*^*S2616*^, the mutation lies within the middle portion of the ankyrin repeats, while the mutation in *mib2*^*S1259*^ and *mib2*^*S1456*^ occurs in the first MIB domain and second RING finger, respectively. It is noteworthy that all three missense mutations result in amino acid changes that include a change in charge. All three mutations affect positions that are highly conserved in fly and vertebrate Mib proteins.

### The hypomorphic alleles *mib2*^*S1456*^ and *mib2*^*S1259*^ survive to adulthood but exhibit defects in adult flight muscles

**Fig 5:**
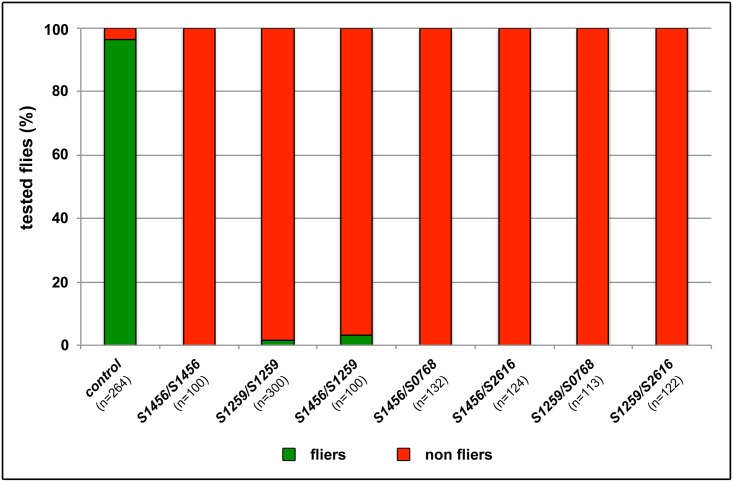
Hypomorphic *mib2* mutants and various allelic combinations are flightless. As compared to EMS control flies, homozygous *mib2*^*S1456*^ and *mib2*^*S1259*^ mutant flies, and those corresponding to different allelic combinations are essentially flightless.

**Fig 6:**
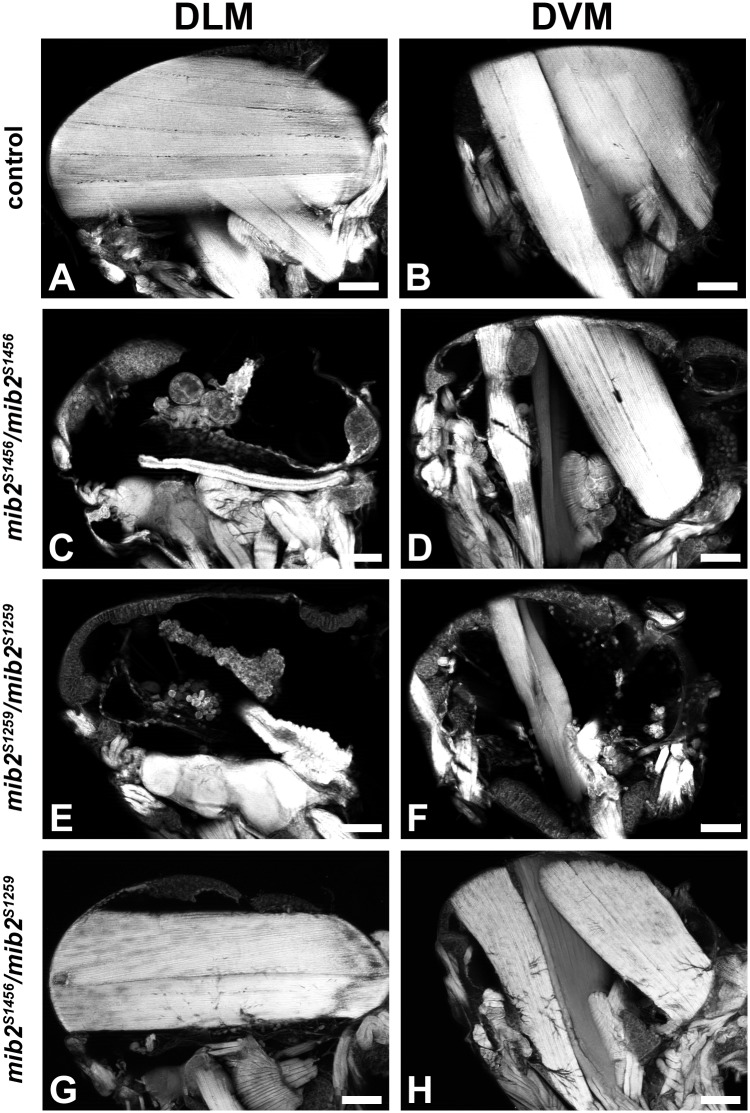
Hypomorphic *mib2*^*S1456*^ and *mib2*^*S1259*^ alleles show defective development of the adult indirect flight muscles. Phalloidin-stained thoraces from adult progeny were subjected to sagittal sectioning with a vibratome. In A, C, E and G, the focus is on the dorsal longitudinal muscles (DLMs), whereas the focus is on the dorso-ventral muscles (DVMs) in B, D, F and H. Shown are representative examples for each indicated genotype (n≧30 hemi-thoraces). In contrast to the control (A), a total loss of the DLMs is observed in homozygous *mib2*^*S1456*^ (C) and *mib2*^*S1259*^ adults (E). In trans-heterozygous *mib2*^*S1456*^/*mib2*^*S1259*^ mutants, there is partial loss of the DLMs (G, showing one of the least severe examples; residual DLMs were present in 35 out of 70 hemi-thoraces). A variable loss of the DVMs is seen in homozygous *mib2*^*S1456*^ (D) and *mib2*^*S1259*^ (F) mutants when compared to the control (B). Trans-heterozygous *mib2*^*S1456*^/ *mib2*^*S1259*^ mutants only exhibit mild DVM defects. (Scale bar = 100μm).

Homozygous *mib2*^*S1456*^ and *mib2*^*S1259*^ mutant flies walk and climb normally, but they are flightless (Fig 5). Adult progeny from trans-heterozygous combinations are also flightless. Moreover, both *mib2*^*S1456*^ and *mib2*^*S1259*^ adults display a characteristic “held-up” (or occasional “held-down”) wing phenotype that could also be observed in trans-allelic combinations with each other or with strong *mib2* mutant alleles. This wing-associated anomaly suggested the existence of thoracic muscle defects. An initial examination of scalpel-generated hemi-thoraces from control and mutant flies revealed that the dorsal longitudinal muscles (DLMs) are often absent in both homozygous *mib2*^*S1456*^ and *mib2*^*S1259*^ mutant flies. For a more detailed analysis, whole thoraces were stained with phalloidin (for F-actin) and sagittal sectioning through each thorax was done with a vibratome. Representative sagittal sections at the level of the DLMs or dorso-ventral muscles (DVMs) are shown in Fig 6. In EMS controls, each hemi-thorax displays six DLMs (Fig 6A). Most homozygous *mib2*^*S1456*^ and all homozygous *mib2*^*S1259*^ mutant thoraces exhibit a total absence of DLMs (Fig 6C and 6E). Many trans-heterozygous *mib2*^*S1456*^*/mib2*^*S1259*^ mutant thoraces also show a total loss of the DLMs, although we also observed thoraces in which DLMs were partially maintained (Fig 6G). With respect to the three sets of DVMs, we observed a differential loss of these muscles in the homozygous *mib2*^*S1456*^ versus the homozygous *mib2*^*S1259*^ mutant thoraces. The *mib2*^*S1259*^ mutants exhibit consistently a more extensive loss (compare Fig 6B, 6D, and 6F), which was also observed in thoraces of *mib2*^*S1259*^*/Df(2L)Exel8039* flies (data not shown). By contrast, only mild DVM defects are observed in trans-heterozygous *mib2*^*S1456*^*/mib2*^*S1259*^ mutant thoraces (Fig 6H). Taken altogether, the mutant phenotype of the hypomorphic alleles indicates that Mib2 function is also required for the formation of adult flight muscles, more strictly for the DLMs than the DVMs, and underscores the functional importance of the MIB domains and RING fingers in this process.

### *In vivo* imaging reveals a requirement of *mib2* in the maintenance of larval DLM templates during early metamorphosis

**Fig 7:**
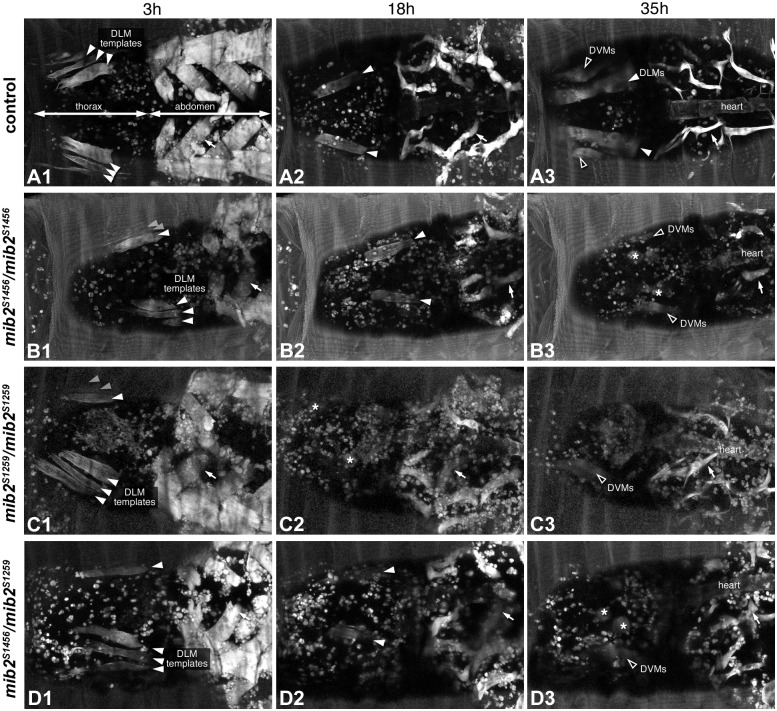
Representative still images from live movies of *mib2* alleles during metamorphosis. Dorsal or dorsolateral views of live pupae carrying *Mhc-tau*::*GFP* to label muscles and developing at 22°C. Images were taken from S1, S3, S4 and S5 Movies. For comparison of the different genotypes, time points are given on a relative scale with t = 0h marking the easily recognizable onset of abdominal compaction, which occurs concurrently with head eversion and appearance of the thorax at ~8h APF at 25°C. (A1-3) Normal development in the control (3h, 18h and 35h after compaction at 22°C; corresponding to approximately 10h, 22h and 35h APF at 25°C, respectively). (B1-3) Live imaging of a homozygous *mib2*^*S1456*^ pupa with degeneration of developing DLMs at 35h after abdominal compaction (asterisks). DVMs (empty arrowheads) are still present. (C1-3) Live imaging of a homozygous *mib2*^*S1259*^ pupa with degeneration of developing DLMs at 18h after abdominal compaction. DVMs are still formed. (D1-3) Live imaging of a trans-heterozygous *mib2*^*S1259*^/*mib2*^*S1456*^ pupa with abnormal DLM development, as seen in *mib2*^*S1456*^ homozygous pupae. Thin arrows label temporarily persistent abdominal dorsal muscles that are derived from larval dorsal acute muscles.

The lack of DLMs in *mib2*^*S1456*^ and *mib2*^*S1259*^ mutants could result from muscle degeneration or defects that occur during DLM formation, which includes aberrant histolysis of the larval templates. Initial analysis indicated that homozygous *mib2*^*S1259*^ or *mib2*^*S1456*^ mutant P7 pupae (corresponding to ~43–47 APF at 25°C; [29]) have a total loss of the DLMs (data not shown). To discriminate between the aforementioned possibilities and to determine the time point during which defects in DLM development first become apparent, we performed non-invasive live imaging of *mib2* hypomorphic alleles and wild-type controls, all of which carry a *Mhc-tau*::*GFP* transgene to label the muscle tissues, during pupal stages (S1–S5 Movies and relevant still images in Fig 7; n≧3 pupae per genotype). The period of observation spans the early steps of DLM formation, including the relocation of larval DLM templates from the anterior to the area of the developing adult thorax, larval muscle histolysis, fusion of persistent larval DLM templates with AMP-derived myoblasts, myotube splitting, attachment and compaction, as well as myofibrillogenesis and sarcomerogenesis in the nascent DLM fibers. This period comprises pupal stages P3-P8, which correspond to ~5-58h APF at 25°C [6, 16]. As seen in the control pupae (S1 and S2 Movies, Fig 7A), most larval body wall muscles are histolyzed during early metamorphosis. In agreement with published observations, we also see two exceptions from this fate, namely the persistent abdominal dorsal acute muscles (thin arrows in Fig 7), which appear to be remodeled during metamorphosis and presumably degenerate at some point after eclosion, and the thoracic oblique muscles, which serve as larval templates for the DLMs [15, 34, 35]. *In vivo* imaging of *mib2*^*S1456*^ (S3 Movie, Fig 7B), *mib2*^*S1259*^ (S4 Movie, Fig 7C) and *mib2*^*S1259*^/*mib2*^*S1456*^ (S5 Movie, Fig 7D) pupae shows that larval muscle histolysis proceeds normally in hypomorphic mutants. The larval DLM templates are also initially spared in the hypomorphic mutants. After abdominal compaction (corresponding to ~8h APF at 25°C; [29]), the persistent larval templates are found at their normal location in the developing thorax (compare Fig 7B1, 7C1 and 7D1 to 7A1). Subsequently, template splitting initiates at ~5-6h after abdominal compaction at 22°C (our testing conditions) in both controls and mutants. By contrast, DLM development proceeds differently in control and mutant pupae at later stages. The nascent DLMs round up in the mutants, but not in the controls, and eventually disappear during template compaction or, at the latest, at the beginning of myofiber maturation and growth. DLM template disintegration consistently occurred earlier in homozygous *mib2*^*S1259*^ mutant pupae than in *mib2*^*S1456*^ or *mib2*^*S1259*^/*mib2*^*S1456*^ mutants (at ~13-18h versus ~23-35h after abdominal compaction at 22°C, which corresponds to ~18-22h versus ~27-35h APF at 25°C; compare Fig 7C1–7C3 with 7B1–7B3 and 7D1–7D3). It is also noteworthy that persistence of larval abdominal dorsal muscles is not affected and that *de novo* generation of DVMs and dorsal abdominal muscles does take place (see S4 Movie). Altogether, these observations suggest that Mib2 function is particularly critical for developing the DLMs, possibly by protecting the larval muscle templates from disintegration during early metamorphosis. The data further suggest additional Mib2 functions such as maintaining the integrity of the DVMs after they are formed.

## Discussion

Mind bomb1 proteins are E3 ubiquitin ligases that interact with a number of proteins, including Notch ligands as the most prominent targets, and proteins involved in diverse processes such as cell death, neuronal morphogenesis, vesicle transport and centriole biogenesis (e.g. [19, 23, 36–39]). Much less is known about the functions of Mib2. It was demonstrated that vertebrate Mib2 can act as a ubiquitin ligase for Notch ligands in cell culture or *in vitro* systems [24, 25]. However, mouse and zebrafish Mib2 were also shown to be dispensable for Notch signaling-dependent processes and embryonic development [40, 41]. Moreover, research in *Drosophila* revealed that Mib2 has Notch signaling-independent functions during embryonic myogenesis that cannot be compensated by Mib1. These embryonic functions comprise a major RING domain-independent function that ensures muscle integrity and a minor RING domain-dependent function that supports efficient muscle fusion [17, 18].

### Mib2 function requires the presence of intact ankyrin repeats

Our *mib2* mutant analysis combined with data from our structure-function studies strongly suggest that the ankyrin repeats comprise the most critical structure in the Mib2 protein. We observed null-like phenotypes in rescue experiments with ankyrin repeat deletions. Although we cannot rule out the possibility that the deletion of these repeats affects the stability or global folding of the mutant protein, we also observed these same phenotypes in two newly isolated alleles, *mib2*^*S0768*^ and *mib2*^*S2616*^, in which the ankyrin repeats are affected (this study). The *mib2*^*S0768*^ allele harbors a nonsense mutation at the end of the fourth ankyrin repeat, leading to a truncated protein that lacks the C-terminal half of the repeat region and the RING domains; this mutation is similar to previously characterized amorphic alleles [17, 18]. The importance of the ankyrin repeats becomes more evident by the missense mutation in *mib2*^*S2616*^ (D564N), which specifically hits a conserved residue in the fourth ankyrin repeat. Taken altogether, the data obtained from phenotypic analysis in embryos demonstrate that the ankyrin repeats are essential for the RING domain-independent functions that are critical for the stabilization of the embryonic/larval musculature. In a wide spectrum of proteins, ankyrin repeats are implicated in mediating protein-protein interactions with target molecules [32]. Therefore, it is possible that the ankyrin repeats in Mib2 may be required in building scaffolds for muscle-specific complexes.

### Different contributions of N-terminal and RING domains to Mib2 function during embryonic myogenesis

Based upon our findings with HERC2/ZZ and MIB domain deletion rescue constructs we suggest that the major Mib2 function in supporting late embryonic/early larval muscle integrity is executed in conjunction with its MIB domains, but not with individual HERC2 and ZZ domains. The relatively mild phenotypes obtained in rescue experiments with HERC2- or HERC2/ZZ-deleted forms of Mib2, which feature a very limited number of unfused myoblasts along with normal muscle fibers, are very similar to those reported for RING domain deletions [17, 18]. It is therefore conceivable that the N-terminal HERC2/ZZ domains might contribute to the same processes as the Mib2 RING domains in that both could be involved in supporting optimal fusion via ubiquitination of particular substrates. HERC2 domains are indeed usually found together with RING or HECT-type E3 ubiquitin ligase domains in Mib, HERC2 and HECTD1 proteins. Several publications have also provided evidence that in *Drosophila* and vertebrate Mib1, the C-terminal RING domain and the N-terminal portion, including HERC2/ZZ/HERC2 and MIB-specific domains, are functionally linked during the ubiquitination and internalization of Notch ligands, such as Delta and Serrate or Jagged [19, 23, 24, 42]. In these studies, the N-terminal domains play an essential role in substrate recognition by binding to particular regions in the intracellular portion of Notch ligands; these observations were further supported by a structural analysis of N-terminal domains of Mib1 with a conserved Notch ligand fragment [43, 44]. Furthermore, McMillan and colleagues showed that the HERC2/ZZ/HERC2 (“MZM module”) and MIB domains of Mib1 bind to different epitopes on a Notch ligand substrate; thus, they proposed a model in which these domains act as independent substrate recognition modules [44].

Our rescue experiments showed a significant decrease in the ability to maintain proper somatic muscle and gut morphology upon deletion of the MIB-specific but not the HERC2 domains. Although we can detect robust expression of the MIB domain-deleted constructs (with ectopic expression levels at least as high as that of full-length Mib2), it is possible that folding or cellular distribution of the protein variants could be different. However, given the modular nature of substrate recognition as discussed above, we propose that the HERC2/ZZ/HERC2 and MIB domains of *Drosophila* Mib2 also act independently to recognize distinct epitopes on different molecules in order to execute multiple functions. Overall our data suggest that at least one of the MIB domains together with the ankyrin repeats is essential for functions needed to stabilize the embryonic musculature, whereas the HERC2 domains, like the RING fingers, could be connected to a subset of ubiquitination-dependent Mib2 functions that do not affect muscle integrity in the embryo. So far, targets that are directly ubiquitinated by Mib2 have not yet been identified, although a couple of candidates can be predicted based on indirect evidence. One candidate that was proposed in the context of myoblast fusion is the Gli-like transcription factor Lame duck (Lmd). Ubiquitinated Lmd is specifically expressed in fusion-competent myoblasts and appears to be degraded in a *mib2*-dependent manner in somatic and visceral muscles after myoblast fusion [18, 45]. In *mib2* mutant embryos, Lmd appears to be ectopically stabilized in maturing myotubes after myoblast fusion where it might interfere with ongoing fusion or fiber differentiation processes [18, 45]. However, in contrast to the postulated structural functions of Mib2 that are mediated via the ankryrin and MIB domains, the presumed E3 ubiquitin ligase-dependent activities of Mib2 appear to contribute relatively little to its major function in maintaining muscle integrity during late embryogenesis [17, 18].

### *Drosophila* Mib2 is a multifunctional protein with distinct roles during embryonic and adult myogenesis

Two hypomorphic alleles newly isolated in this work point toward additional post-embryonic functions of Mib2. These alleles, *mib2*^*S1259*^ and *mib2*^*S1456*^, were found to contain point mutations in the MIB and RING domains, respectively. In contrast to the *mib2* mutations affecting ankyrin repeats, these mutations have only minor impact on embryonic development allowing the flies to survive to adulthood. Strikingly, these mutations strongly impair functions of Mib2 during adult myogenesis, leading to an inability to fly and a frequent “wings held-up” phenotype. Such phenotypes are usually connected with mutations in genes that affect flight muscle development, sarcomeric structure or innervation [46, 47]. Our present data demonstrate that in these *mib2* hypomorphic alleles, the phenotypes are essentially caused by degeneration of nascent DLMs during metamorphosis and by DVM defects to a lesser degree. The severe loss of these muscles can fully explain the visible phenotypes of the adults. Our data are also consistent with observations made in adults after RNAi-mediated down-regulation of *mib2* using the post-embryonic driver *1151-GAL4* [18].

Notably, the phenotype of our hypomorphic *mib2* mutants and the timing of DLM template loss are reminiscent of phenotypes described for specific hypomorphic mutants of *stripe*, a transcription factor-encoding gene expressed in epidermal cell types that regulates muscle attachments in embryos and during metamorphosis [34, 48–50]. It is therefore conceivable that Mib2 might also be vital for the nascent DLM fibers to form stable attachments, which appear to be interconnected with myofibrillogenesis [51]. Mib2 could act in several ways. First, Mib2 could be directly involved in setting up cell contacts, although in the embryo no influence on integrin-marked muscle attachment sites was found in *mib2* hypomorphs (this work) and the defects in amorphs are presumed to be secondary [17]. Second, Mib2 could provide linkage to intracellular filaments or myofibrills; alternatively, it could assist in their formation. Of note, Mib2 is localized to Z-discs and to a lesser extent to M-lines of sarcomeres in larval and adult muscles [18, 28]. Furthermore, non-muscle myosin II components, which are also found at Z-discs in larvae, have been co-immunoprecipitated with Mib2 from adult thoraces [18, 52]. Finally, Mib2 could act indirectly to support growth and differentiation of nascent myotubes by influencing vesicle transport or signaling through processes similar to Mib1 in other systems. Data from the *Drosophila* protein interaction map (DPiM), which was obtained from proteome-wide purification of affinity-tagged proteins after expression in cell culture, suggest interactions of Mib2 with several proteins that are either part of the ESCRT (Endosomal Sorting Complexes Required for Transport) machinery or are predicted to have related functions [53]. The ESCRT pathway plays an important role in protein degradation as well as the distribution and down-regulation of cell-surface receptors, including Notch (reviewed in [54]).

### Location of the hypomorphic mutations suggests a ubiquitination-associated role of Mib2 during development of adult flight muscles

The glycine-to-glutamic acid exchange observed in the *mib2*^*S1259*^ allele (G277E) is situated in the first MIB repeat in a surface pocket that was recently shown to be essential for binding of mammalian Mib1 to a particular region of the Notch ligand Jag1 [44]. The G277E mutation causes the first MIB repeat to look more like the second MIB repeat within this region (GHGGW versus GHGEW), which might lower the affinity for specific targets but not generally impair binding. As a result, this mutant form of Mib2 might still interact with most of its embryonic targets but not those in developing DLMs. As discussed above, previous data did not associate Mib2 with Notch signaling-related processes [17, 18]; hence, the existence of other interaction partners appears plausible. Nevertheless, since Notch signaling has to be tightly regulated during IFM formation [55–57], a connection between Mib2 and Notch signaling during flight muscle development remains an interesting possibility that needs to be addressed in future experiments.

The C-terminal-most RING finger of Mib2 and the corresponding RING finger in Mib1 appear to be the most critical for E3 ubiquitin ligase activity. *Drosophila mib1* mutants that lack only the C-terminal-most third RING finger are indistinguishable from other amorphic *mib1* alleles [20, 22], and mutations in the second RING finger of mouse and zebrafish Mib2 eliminate ubiquitin ligase activity in cell culture or *in vitro* [24, 25]. Therefore, the second hypomorphic mutation obtained from our EMS screen, which changes an invariant glycine to an arginine in the C-terminal-most RING finger (G1021R in *mib2*^*S1456*^) is predicted to significantly reduce the ubiquitin ligase activity. Since this mutation results in a similar phenotype as the hypomorphic MIB domain mutation G277E in *mib2*^*S1259*^, we presume that in both cases, DLM formation is abrogated as a consequence of reduced ubiquitination due to impaired ligase activity or substrate recognition. Notably, the trans-heterozygous combination of our hypomorphic *mib2* alleles showed similar but milder defects than both alleles in homozygous condition, which suggests the existence of multimeric complexes in which a mixture of the differently mutated Mib2 molecules could provide some residual activity. This possibility is consistent with the notion that Mib proteins are able to form homo- and hetero-oligomers [25]. Additionally, ankyrin repeats, which could provide protein-protein interaction interfaces as mentioned above, might mediate this interaction [25]. The identity of the critical Mib2 targets during DLM formation remains to be identified. In this regard, our novel viable hypomorphic alleles could serve as excellent tools for future genetic or proteomic approaches.

## Supporting information

S1 Fig Quantification of skeletal muscles fibers in *mib2* mutants with the rescue constructs shown in Fig 1. For each indicated genotype, 25 abdominal hemi-segments (A2-A6) were analyzed for the presence of 14 easily recognizable fiber types (DO3/4, DT1, LT1-4, LO1, SBM, VO1/2, VO4-6). Average fiber numbers are indicated within each column. Asterisks denote significant differences in muscle numbers (**P<0.0005).
(TIF)

S1 Movie Live imaging of normal muscle development during metamorphosis. Dorsal view of a control pupa carrying a *Mhc-tau*::*GFP* from early P4 to P7/8 at 22°C. Time (denoted as h:min:s) is relative to the onset of abdominal compaction (corresponding to ~8h APF at 25°C; for comparison to the APF scale at 25°C, the observed time period at 22°C needs to be divided by a factor of 1.3 to take into account the slower development). Most larval muscles are histolyzed prior to t≈10h, except for specific persistent larval dorsal abdominal muscles and three thoracic oblique muscles per hemi-segment (initially located near the anterior end on the left; arrowheads), which serve as templates for DLM formation. Of the three bilateral DLM templates, only the most medial ones stay in the monitored dorsal region. After template compaction occurring at ~12-26h, the developing DLMs extend and form the mature longitudinal muscles. During the second half of the movie, newly formed DVMs (empty arrowheads) also become recognizable at more lateral positions of the thorax.
(MP4)

S2 Movie Live imaging of normal muscle development during metamorphosis of a second control pupa with a *Mhc-tau*::*GFP* transgene. The non-mutagenized chromosome in this control pupa has a tinC*-GFP transgene that is active in the dorsal vessel. Dorso-lateral view during stages P4-P8 at 22°C. Development proceeds as in S1 Movie. Near the end of this movie, thoracic DLMs (arrowheads) and DVMs (empty arrowheads) as well as the abdominal dorsal longitudinal muscles can be seen.
(MP4)

S3 Movie Live imaging of a homozygous *mib2*^*S1456*^ pupa with a *Mhc-tau*::*GFP* transgene. Time scale and orientation are analogous to the control in S1 Movie. The thoracic DLM templates (arrowheads) are initially present and persist through an early phase of DLM formation, but disintegrate subsequently (at ~30-35h; asterisks). Overall development continues as indicated by the appearance of DVMs (empty arrowheads) in the thorax and beating of the heart in the middle of the abdomen.
(MP4)

S4 Movie Live imaging of a homozygous *mib2*^*S1259*^ pupa carrying *Mhc-tau*::*GFP* and tinC*-GFP transgenes. Time scale and orientation are analogous to the control in S2 Movie. The thoracic DLM templates (arrowheads) persist through an initial phase of DLM formation, but soon disintegrate (at the onset of myotube compaction at ~14-18h; asterisks). Overall development continues as indicated by the appearance of DVMs (empty arrowheads) in the thorax and abdominal dorsal longitudinal muscles as well as beating of the heart.
(MP4)

S5 Movie Live imaging of a trans-heterozygous *mib2*^*S1259*^/*mib2*^*S1456*^ pupa carrying *Mhc-tau*::*GFP* and tinC*-GFP transgenes. The thoracic DLM templates (arrowheads) are initially present, but disintegrate subsequently at a similar time as in *mib2*^*S1456*^/*mib2*^*S1456*^ (at ~28-35h; asterisks). Development continues as in the homozygous mutant pupae.
(MP4)
