# Supplementary figures and images for: Identification of the essential protein domains for Mib2 function during the development of the Drosophila larval musculature and adult flight muscles
Source: PLoS One. 2017 Mar 10;12(3):e0173733. doi: 10.1371/journal.pone.0173733 (PMC5345843; doi:10.1371/journal.pone.0173733)

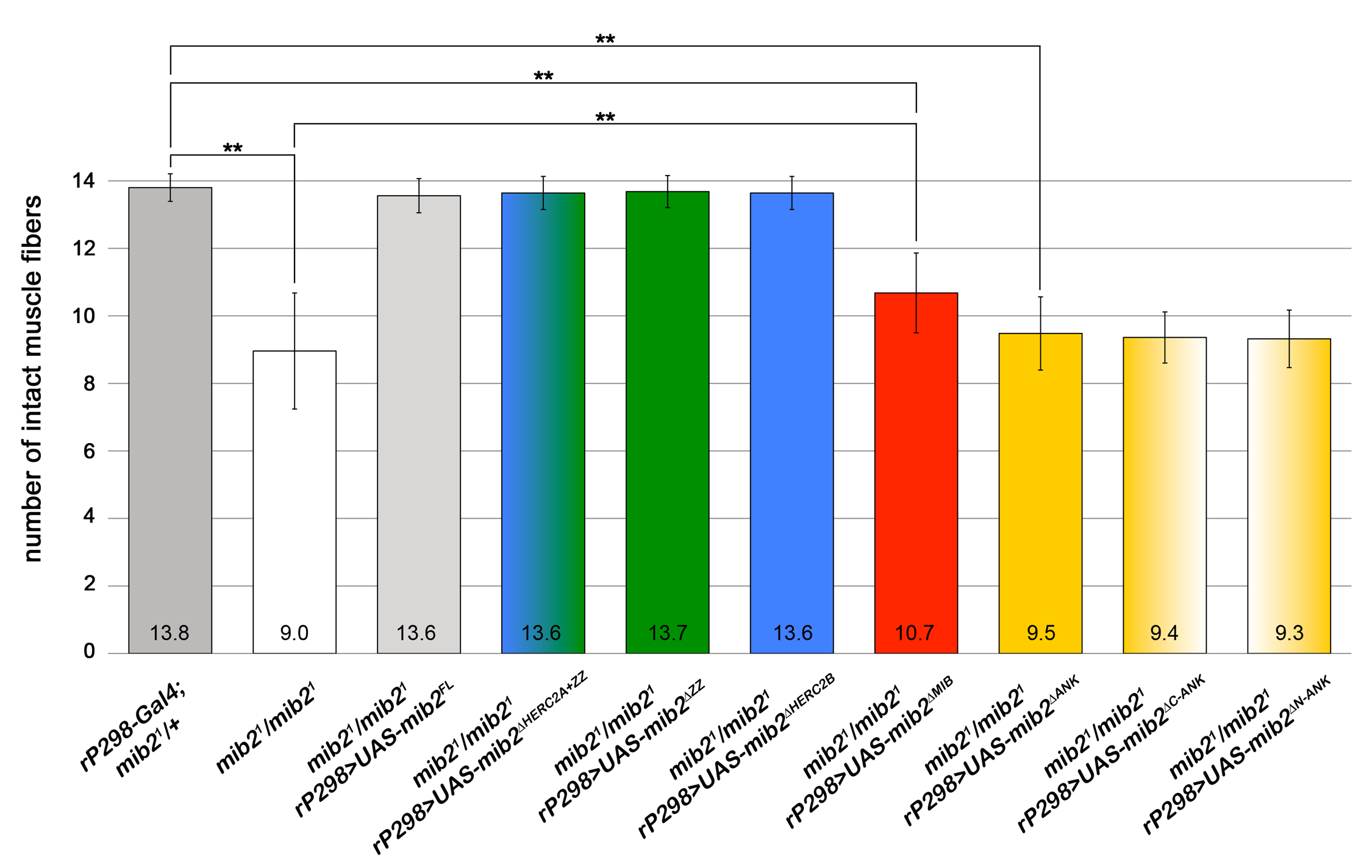

Supplement: S1 Fig — For each indicated genotype, 25 abdominal hemi-segments (A2-A6) were analyzed for the presence of 14 easily recognizable fiber types (DO3/4, DT1, LT1-4, LO1, SBM, VO1/2, VO4-6). Average fiber numbers are indicated within each column. Asterisks denote significant differences in muscle numbers (**P<0.0005). (TIF) [file pone.0173733.s001.tif]
